# Prognostic Factors of Colorectal Cancer: A Comparative Study on Patients With or Without Liver Metastasis

**DOI:** 10.3389/fonc.2021.626190

**Published:** 2021-12-21

**Authors:** Honghua Peng, Guifeng Liu, Ying Bao, Xi Zhang, Lehong Zhou, Chenghui Huang, Zewen Song, Sudan Cao, Shiying Dang, Jing Zhang, Tanxiao Huang, Yuling Wu, Mingyan Xu, Lele Song, Peiguo Cao

**Affiliations:** ^1^ Department of Oncology, the Third Xiangya Hospital of Central South University, Changsha, China; ^2^ The Medical Division, HaploX Biotechnology, Shenzhen, China; ^3^ Department of Radiotherapy, the Eighth Medical Center of the Chinese People’s Liberation Army (PLA) General Hospital, Beijing, China

**Keywords:** colorectal cancer, liver, metastasis, prognosis, chemotherapy, surgery

## Abstract

**Background:**

Radical or palliative surgery with subsequent adjuvant therapy is the routine treatment for stage II/III colorectal cancer(CRC) and some stage IV CRC patients. This study aimed to clarify the prognostic clinicopathological and genetic factors for these patients.

**Methods:**

Fifty-five stage II-IV CRC patients undergoing surgery and adjuvant therapy were recruited, including patients without liver metastasis(5 at stage II, 21 at stage III) and with liver metastasis(29 at stage IV). Genetic alterations of the primary cancer tissues were investigated by whole exome sequencing(WES). Patients were followed up to 1652 days(median at 788 days).

**Results:**

The mutational landscape of primary CRC tissue of patients with or without liver metastasis was largely similar, although the mutational frequency of TRIM77 and TCF7L2 was significantly higher in patients with liver metastasis. Several main driver gene co-mutations, such as TP53-APC, APC-KRAS, APC-FRG1, and exclusive mutations, such as TP53-CREBBP, were found in patients with liver metastasis, but not in patients without liver metastasis. No significant difference was found between the two groups in aberrant pathways. If stage II-IV patients were studied altogether, relapse status, SUPT20HL1 mutations, Amp27_21q22.3 and Del8_10q23.2 were independent risk factors(P<0.05). If patients were divided into two groups by metastatic status, surgery types and Amp6_20q13.33 were independent risk factors for patients without liver metastasis(P<0.05), while TRIM77 mutations were the only independent risk factor for patients with liver metastasis(P<0.05).

**Conclusions:**

Surgery types and Amp6_20q13.33 were independent risk factors for CRC patients without liver metastasis, and TRIM77 mutations were the independent risk factor for CRC patients with liver metastasis.

## Introduction

Colorectal cancer (CRC) is the third most common cancer with the second mortality (1). The five-year survival for stage I, II, III and IV was reported to be approximately 90%, 80%, 30-60% and less than 10% (1). 20%-30% of CRC patients were diagnosed with distal metastasis at the first visit (2, 3). Liver metastasis was the most common distal metastasis and accounted for more than 50% distal metastasis in CRC (2, 3).

Early stage CRC can be treated with endoscopic mucosal resection (EMR), endoscopic submucosal dissection (ESD) or radical surgery, and patients can be cured with high survival rate (4). Patients with advanced CRC or metastatic CRC can be managed by a series of systematic therapy combined with surgery. Radical or palliative surgery with subsequent adjuvant therapy is the conventional method for patients with advanced CRC or patients with liver metastasis (5). Radical surgery with adjuvant therapy is used for patients who have locally advanced CRC with lymph node metastasis and/or solitary liver metastasis that can reach R0 resection (5, 6). Palliative surgery with adjuvant therapy is used for patients with comprehensive local invasion and/or multiple liver metastases (5, 6). Patients with multiple unresectable metastases are recommended for systematic therapy without recommendation for surgery (5–7).

Although surgery with subsequent adjuvant therapy is used widely in CRC treatment, the prognostic and risk factors for patients undergoing the treatment remain to be clarified. It is currently not possible to predict the prognosis of these patients from the genetic alterations of primary cancer tissues due to the lack of effective markers. Although some studies on liver metastatic tissues found that mutations from several driver genes were capable of predicting the prognosis of patients with liver metastasis (8, 9), there are few reports on the correlation between primary cancer tissue genetic alterations and patient survival or prognosis. Meanwhile, undifferentiated treatment may result in unnecessary attempt with no clear expectation of therapeutic response and long-term survival. In order to solve these problems, we performed the first study to investigate the potential prognostic and risk factors for patients underwent surgery with adjuvant therapy. Patients with or without liver metastasis were both studied, the genetic alterations of primary cancer tissues were examined by whole-exome sequencing, the clinicopathological information was collected, and all patients were followed up to 1652 days (median at 788 days). We identified several clinicopathological and genetic alterations that can potentially predict the prognosis and survival for both metastatic and non-metastatic patients, and clarified the risk factors for these patients.

## Methods and Materials

### Ethics, Patients, and Samples

A retrospective cohort study was designed and implemented at the third Xiangya hospital of central south university (Changsha, Hunan province, P. R. China). This research was approved by the Ethics Committee of the third Xiangya hospital of central south university and conducted in accordance with the hospital’s guiding principles. Since retrospective samples were used, written informed consent was waived for the use of the clinical samples. Patient information was kept anonymous for confidentiality. Samples were selected, and tests were performed based on the diagnosis, availability of clinical and follow-up information, and quality control of the sequencing (**Table 1**).

**Table 1:** The clinicopathological information of all patients recruited in this study.

Clinicopathological factors	Subgroups	Number of subjects (%)
Gender		
	Female	26 (47.3%)
	Male	29 (52.7%)
Age		
(median:56)	<56	25 (45.5%)
	≥56	30 (54.5%)
Stage		
	Stage II	5 (9.1%)
	Stage III	21 (38.2%)
	Stage IV	29 (52.7%)
Surgery		
	Palliative	19 (34.6%)
	Radical	36 (65.4%)
Cancer type		
	Rectal cancer	44 (80.0%)
	Colon cancer	11 (20.0%)
Relapse	Stage II-III	7/26 (26.9%)
(followed up to 1652 days, median:788 days)	Stage IV	19/29 (65.6%)

The inclusion criteria included adults over 18 years old, patients with complete clinicopathological information, and a confirmed diagnosis of CRC (including endoscopy, magnetic resonance imaging (MRI), or computed tomography (CT)), and subsequent pathological examinations and immunohistochemical staining. Patients treated with surgery with subsequent adjuvant therapy (including chemotherapy, radiotherapy or both combined) were included, and only patients who had tissue samples available for testing were included. The exclusion criteria included those patients not treated by surgery with subsequent adjuvant therapy, a history of cancers other than CRC, unavailability of samples or follow-up information, or samples unable to pass the quality controls in sequencing. Thus, a cohort of 55 patients (**Table 1**) with confirmed CRC were available for study and all of them were enrolled in sequencing study. A summary of the clinicopathological information for all patients is listed in **Table 1**.

### DNA Extraction and Quantification

WES was performed with both tumor tissue samples and peripheral blood leukocytes (PBLs). DNA from peripheral blood leukocytes was used as the control for tumor tissue mutation calling. For the tumor tissue formalin fixation and paraffin embedding (FFPE) samples, ten 5 μm tissue sections were taken for DNA extraction, using the QIAamp DNA FFPE Kit (QIAGEN, Valencia, CA, USA), and following the manufacturer’s instructions. RelaxGene blood DNA system (Tiangen Biotech) was used to extract genomic DNA from peripheral blood lymphocytes (PBLs). The quality control for the DNA was achieved using Qubit 2.0 (Thermo Fisher Scientific), following the manufacturer’s instructions.

### Library Construction, Whole-Exome Sequencing, and Data Processing

The fragmented genomic DNA underwent end-repairing, A-tailing and ligation, and then was sequentially completed with indexed adapters, followed by size selection using Agencourt AMPure XP beads (Beckman Coulter Inc., Brea, CA, USA). The DNA fragments were used for library construction with the KAPA Library Preparation kit (Kapa Biosystems, Inc., Wilmington, MA, USA) according to the manufacturer’s protocol. Seven to eight polymerase chain reaction (PCR) cycles, depending on the amount of DNA used, were performed on pre-capture ligation-mediated PCR (Pre−LM−PCR) Oligos (Kapa Biosystems, Inc.) in 50μl reactions. The DNA sequencing was performed using a WESPlus gene panel (an upgraded version of the standard whole exome sequencing (WES), HaploX Biotechnology) for tumor tissue sequencing on the Illumina Novaseq 6000 system according to the manufacturer’s instructions.

Sequencing data were filtered by fastp and aligned to the hg19 genome (GRch37) using Burrows Wheeler Aligner (BWA). SAMtools was used to sort the BAM files and perform duplicate marking. The Gencore version 0.12.0 (https://github.com/OpenGene/gencore) was used to remove duplicate reads. Somatic variants were determined using MuTect2. Somatic mutations were detected by bioinformatics analysis against the reference genome. New panel of normal (PON) created by in-house healthy individual using GATK. ANNOVAR was performed to annotate the Variant Call Format file obtained in the previous step. The tumor mutation burden (TMB) was identified as the total number of incorrect codings, base substitutions, and insertions and deletions in somatic cells per one million bases. The TMB was calculated by dividing the total number of tissue non-synonymous SNV and INDEL variations (with allele frequencies ≥ 5%) by the size of the coding region covered by the WES panel.

### Statistical Analysis

All charts were generated, and data analyses performed using Graphpad PRISM 5.0 or the R statistical software package (https://www.r-project.org/). The Chi-square test was used to compare the ratios or percentages between two groups. The Kaplan-Meier survival analysis was performed, and the resulting curves were plotted with the R software and compared using the Log-rank (Mantel-Cox) test. The mutational landscape figures were plotted using the ‘Complex Heatmaps’ package included in the R statistical software package. Univariate and multivariate Cox regression analyses were performed to investigate the risk factors using SPSS 17.0. P<0.05 was considered to be statistically significant.

## Results

### Mutational Landscape of Primary Cancer in CRC Patients With or Without Liver Metastasis

**Figure 1:**
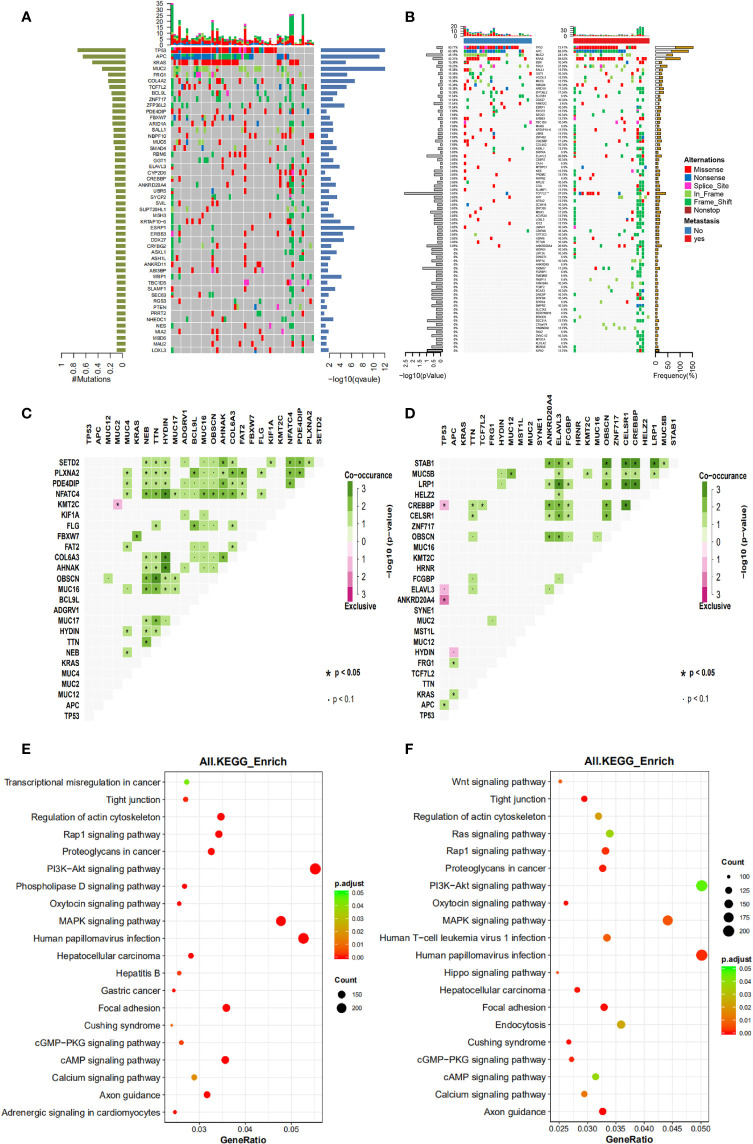
The mutational spectrum, co-mutations, exclusive mutations and pathway analysis of CRC patients with or without liver metastases. **(A)** the mutational spectrum of all patients involved in this study, ranked by mutational frequency. **(B)** the mutational spectrum of patients without liver metastases (left panel) or patients with liver metastases (right panel). **(C)** the significant co-mutations and exclusive mutations of patients without liver metastases. **(D)** the significant co-mutations and exclusive mutations of patients with liver metastases. **(E)** the KEGG pathway enrichment analysis of patients without liver metastases. **(F)** the KEGG pathway enrichment analysis of patients with liver metastases.

The mutational landscape of primary cancer lesions in CRC patients with or without liver metastasis was investigated and compared. The landscape of all involved patients is shown in **Figure 1A** and the individual landscape of patients with or without liver metastasis is shown in **Figure 1B**. It can be seen that the top mutated genes included TP53, APC, KRAS, MUC2 and FRG1 when all patients were examined (**Figure 1A**), and this was also true when patients with (**Figure 1B** right panel) or without (**Figure 1B** left panel) liver metastasis was examined individually, suggesting no significance between the two groups in top mutated genes. Analysis of tumor mutational burden (TMB) found no significant difference between the two groups. The mutated genes were compared in parallel in **Figure 1B**, with frequency for both groups labeled. No significant difference was found on the frequency of most genes, however, TCF7L2 (P=0.003) and TRIM77 (P=0.05) single nucleotide variation/insertion and deletion (SNV/INDEL) mutations showed significant difference between the two groups (**Supplementary Table 1**). Chi-square test showed that the mutational frequency of these two genes was significantly higher in patients with liver metastasis than those without, and their frequency in patients without liver metastasis was very low. This observation suggests that TCF7L2 and TRIM77 mutations could be used as genetic indicators for liver metastasis.

Co-mutations and exclusive mutations were also examined. It can be observed from **Figure 1C** (patients without liver metastasis) and **Figure 1D** (patients with liver metastasis) that huge difference existed in co-mutations or exclusive gene pairs between the two groups of patients. For example, for main driver genes such as APC, TP53 and KRAS, there were no significant co-mutations for TP53 and APC, and only one co-mutation for KRAS (with FBXW7) was found in patients without liver metastasis, and only one exclusive pair (with MUC2 or KMT2C) was found. In contrast, one co-mutation for TP53 (with APC), and two co-mutations for APC (with KRAS or FRG1) were found in patients with liver metastasis, and three exclusive pairs were found with TP53 (with CREBBP, ELAVL3 or ANKRD20A4) and one exclusive pair was found with APC (with HYDIN). These observations suggest that although no significant difference was found in the mutational landscape between the two groups, and intragroup mutation correlation differed substantially.

The aberrant pathways of the two groups of patients were further examined. Results of Kyoto Encyclopedia of Genes and Genomes (KEGG) pathway enrichment analysis are shown here to illustrate the status of involved pathway of the two groups. It can be seen that patients without liver metastasis (**Figure 1E**) shared major aberrant pathways with patients with liver metastasis (**Figure 1F**), including human papillomavirus infection, MAPK signaling pathway, PI3K-Akt signaling pathway, and focal adhesion, etc. Difference existed in some pathways or functions with small amount of mutations, including Wnt signaling pathway, endocytosis, phospholipase D signaling pathway, and Hippo signaling pathway, etc. These observations suggested that no substantial but small differences were found between the two groups in aberrant signaling pathway.

### The Prognostic Factors for All Stage II-IV CRC Patients Underwent Surgery and Adjuvant Therapy

**Figure 2:**
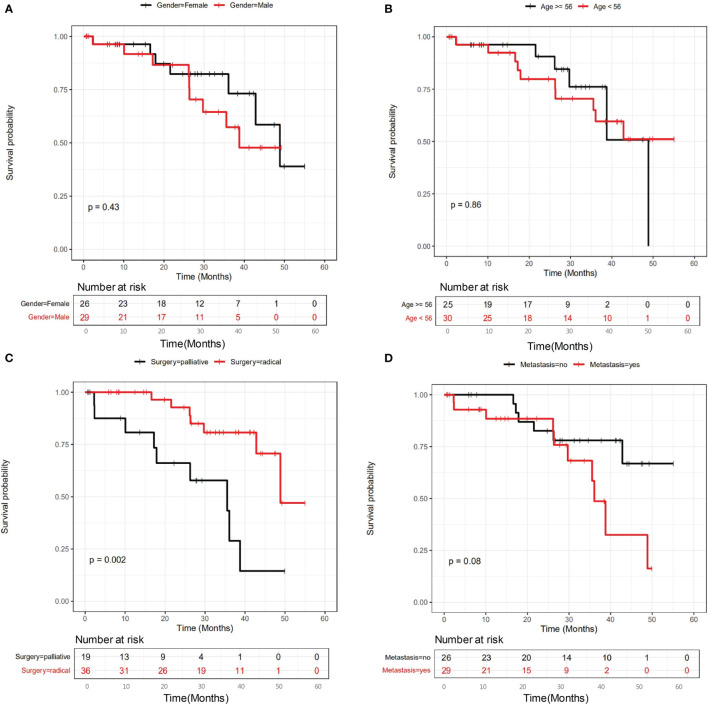
The influence of clinicopathological factors on the prognosis of all patients involved in this study. The influence of gender, age, surgery types and metastasis were shown in panel **(A–D)**, respectively.

The clinicopathological and genetic factors that could potentially affect the patient long-term prognosis were investigated in detail. Patients were followed up to 1652 days (median at 788 days). All stage II-IV patients were first investigated together to identify the prognostic factors for locally advanced and metastatic CRC as a whole. **Figure 2** shows that gender (**Figure 2A**) and age (median at 56 years old) (**Figure 2B**) did not stratify the patient long-term prognosis, while surgery types (radical or palliative) (**Figure 2C**) and metastatic status (**Figure 2D**) may stratify the patient prognosis. It appeared that patients underwent radical surgery exhibited significantly better survival than those underwent palliative surgery (P=0.002), and patients with no liver metastasis exhibited a trend of better survival than those with liver metastasis (P=0.08).

**Figure 3:**
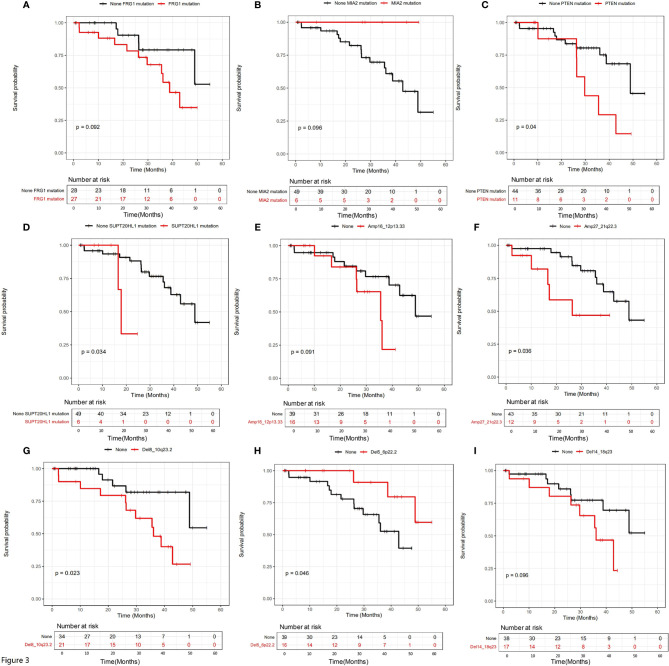
The influence of genetic variations on the prognosis of all patients involved in this study. The influence of a series of SNV/INDEL mutations were shown in **(A–D)**, the influence of CNV variations including a series of amplifications and deletions were shown in **(E–I)**. Gene names were labeled.

We further examined the relationship between mutational status and patient survival. All genes with statistically significant number of mutations were examined, and those showing significant or close to significant stratification between mutated and unmutated groups are shown in **Figure 3**. It is clear that SNV/INDEL mutations of FRG1 (**Figure 3A**, P=0.092), MIA2 (**Figure 3B**, P=0.096), PTEN (**Figure 3C**, P=0.04) and SUPT20HL1 (**Figure 3D**, P=0.034) may be prognostic factors for stage II-IV patients, and copy number variation (CNV) changes including Amp16_12p13.33 (**Figure 3E**, P=0.091), Amp27_21q22.3 (**Figure 3F**, P=0.036), Del8_10q23.2 (**Figure 3G**, P=0.023), Del5_6p22.2 (**Figure 3H**, P=0.046), and Del14_18q23 (**Figure 3I**, P=0.096) may be prognostic factors for stage II-IV patients.

Both univariate and multivariate Cox regression analyses were performed to identify the potential risk factors (**Supplementary Table 2**). Factors with statistical significance in univariate analysis were further examined in multivariate analysis. **Supplementary Table 2** show that surgery type, cancer stage, relapse status, SUPT20HL1 and PTEN mutation status, and CNV variations including Amp27_21q22.3, Del8_10q23.2, Del5_6p22.2 were risk factors in univariate analysis, in which relapse status, SUPT20HL1 mutational status, Amp27_21q22.3 and Del8_10q23.2 were independent risk factors for stage II-IV CRC patients in multivariate analysis.

### The Prognostic Factors for CRC Patients With or Without Liver Metastasis Underwent Surgery and Adjuvant Therapy

**Figure 4:**
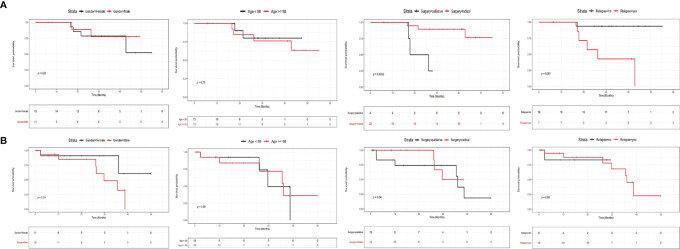
The influence of clinicopathological factors on the prognosis of CRC patients with or without liver metastases. The influence of gender, age, surgery types and relapse status for patients without liver metastasis was shown in **(A)**, and the influence of these factors for patients with liver metastasis was shown in **(B)**.

In order to clarify the prognostic factors for CRC patients with or without liver metastasis, we divided all involved CRC patients into two groups based on metastatic status, and investigated the correlation between clinicopathological factors or mutational status and their survival. **Figure 4** shows the influence of clinicopathological factors on the prognosis of patients without liver metastasis (**Figure 4A**) or with metastasis (**Figure 4B**). It can be clearly seen that gender and age were not prognostic factors for patients without liver metastasis (**Figure 4A**), while surgical types and relapse status were prognostic factors. Patients with radial surgery exhibited significantly better survival than those with palliative surgery (P=0.0032), and patients with no relapse exhibited significantly better survival than those with relapse (P=0.001). In contrast, none of the examined factors showed significant results in patients with liver metastasis (**Figure 4B**), suggesting that the prognosis of these patients cannot be stratified by gender, age, surgical types or relapse status.

**Figure 5:**
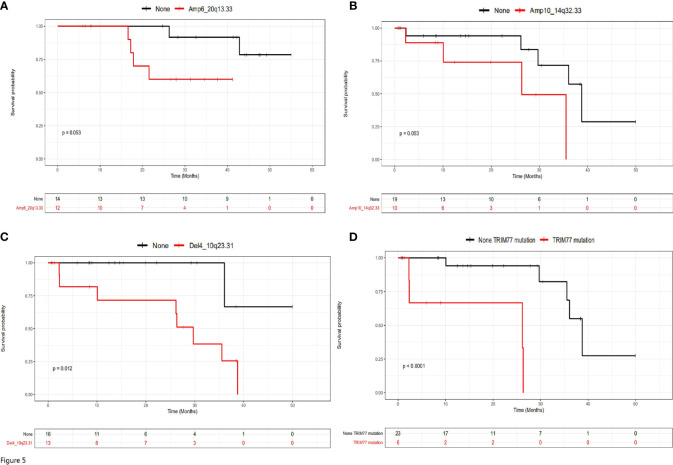
The prognostic genetic variations for patients with or without liver metastasis. **(A)** Amp6_20q13.33 was the only significant prognostic genetic variation for patients without liver metastasis. **(B–D)** Amp10_14q32.33, Del4_10q23.31 and TRIM77 mutations were significant prognostic genetic variations for patients with liver metastasis.

The relationship between mutational status and prognosis were further examined in patients with or without liver metastasis separately (**Figure 5**). Amp6_20q13.33 was the only factor with a trend of statistical significance in patients without liver metastasis (**Figure 5A**, P=0.053), while Amp10_14q32.33 (P=0.053), Del4_10q23.31 (P=0.012) and TRIM77 mutations (P<0.0001) were three variations with statistical significance in patients with liver metastasis (**Figures 5B–D**). Therefore, these four variations appeared to stratify the prognosis of the two groups of patients under the current therapeutic regimes.

Both univariate and multivariate Cox regression analysis were performed for patients without liver metastasis (**Supplementary Table 3**) or with liver metastasis (**Supplementary Table 4**). It can be seen that although surgery types, relapse status and Amp6_20q13.33 were risk factors for patients without liver metastasis, only surgery types and Amp6_20q13.33 were independent risk factors for these patients (**Supplementary Table 3**). In contrast, although Amp10_14q32.33, Del4_10q23.31 and TRIM77 were risk factors for patients with liver metastasis, only TRIM 77 was independent risk factor for these patients (**Supplementary Table 4**).

## Discussion

### Difference in Mutational Landscape of CRC With or Without Liver Metastasis, and Their Indications

Stage II-IV colorectal cancer (CRC) patients contain a mixed population of patients without metastasis but with advanced primary tumor, patients with lymph node metastasis and patients with distal metastasis. Radical surgery aims to achieve R0 resection with adjuvant therapy to prevent metastasis or relapse. Palliative surgery aims to reduce the symptoms and prolong the survival of patients. In this study, we focused on this population and investigated the prognostic and risk factors that may affect the patient survival. There are few reports so far investigating the mutational discrepancy of primary CRC tissues between patients with and without liver metastasis while many studies focused on the concordance and differences between primary and liver metastatic tissues (3, 10–14). This is because the mutational profile between patients with and without liver metastasis were shown to be largely identical across different stages of CRC (3, 10–14), while difference in mutation can only be identified by large panel of next-generation sequencing (NGS), such as WES, with relatively large number of samples. The intratumoral heterogeneity of CRC was shown to be much less than other cancers such as lung cancer (15, 16), making it more difficult to identify differentially mutated genes. Therefore, report on differential mutational frequency of TCF7L2 and TRIM77 in this study represented the first report on such difference between patients with and without liver metastasis.

Furthermore, the huge differences in co-mutations and exclusive mutations between patients with and without liver metastasis are of great interest. Although the mutational landscape between the two groups appeared to be largely similar, the huge differences suggest that the profile of mutation correlation was distinct, which may be associated with the genetic origin and driving factors of liver metastasis. The roles of co-mutations and exclusive mutations in CRC carcinogenesis and liver metastasis have not been well understood. Previous observations suggested that KRAS mutation status was highly homogeneous between areas of the primary tumor and the corresponding metastasis of colorectal adenocarcinomas, and KRAS mutation was associated with more rapid and aggressive metastatic behavior of colorectal liver metastases (13, 17). It was also suggested that the RAS/TP53 co-mutation was independently associated with increased risk of recurrence in CRC patients with resection of liver metastasis (2). In addition, co-mutation of BRAF and APC generated an extremely aggressive neoplastic phenotype that is associated with poor outcome in CRC patients (18). It could be suggested that co-mutations composed of driver genes such as TP53-APC, APC-KRAS and APC-FRG1, and exclusive mutations such as TP53-CREBBP may represent specific liver metastatic molecular subtypes in primary tumor tissues, and these subtypes may be more inclined to develop liver metastasis than those without these co-mutations or exclusive mutations.

### Prognostic and Risk Factors for Patients With or Without Liver Metastasis, and Their Roles in Prognosis Stratification

Investigation on the prognostic and risk factors for patients underwent surgery with adjuvant therapy was the focus of this study. However, the prognostic and risk factors of stage II/III patients underwent radical surgery may be different to stage IV patients underwent palliative surgery, and this was why we examined all patients first and then divided them into two groups by metastatic status. The results from analysis proved our speculation. It was quite interesting to find that the prognostic and risk factors derived from all patients had little overlap with the factors derived from patients with or without liver metastasis alone, and surgery types were the only shared factor. Other factors, including mutational status, copy number changes, metastatic status or relapse status, did not exhibit common predictive capability. These observations suggest that the tumor status of patients did affect the predicting efficacy, and the prognostic and risk factors examined were quite sensitive to various conditions. Therefore, different stages of patients with various status of tumor size, tumor invasion, lymph node metastasis, and distal metastasis should not be combined together in survival or prognostic analysis, as the results could be misleading and lack of practical significance in patient prognostic or risk stratification.

In patients with no liver metastasis, we found that surgery types, relapse status were significant prognostic factors, in which surgery types were independent risk factors. Indeed, since these patients had no distal metastasis, radical surgery would be beneficial in terms of prognosis and long-term survival. However, if radical surgery was not possible, the risk of relapse would be high and the patients would have poor prognosis. This is why both surgery types and relapse status were significant prognostic factors in this group. However, it appeared that relapse was not an independent risk factor but surgery types were independent because relapse was closely related to the manner of surgery. In contrast, the surgery types and relapse status were not prognostic factors for patients with liver metastasis. This could be due to the difficulty in performing radical surgery on primary tumors in patients with liver metastasis, and even the primary tumors were completely removed (R0 resection), the remaining liver metastasis was still a factor for poor prognosis and survival. Therefore, whether or not the primary tumors were completely removed did not affect the prognosis of patients with liver metastasis. The same rationale also applied to the relapse status, in which no difference in long-term survival was observed in patients with relapse or not, as the prognosis of patients with liver metastasis was poor regardless of relapse status.

In this study, we identified a few novel genetic variations with significant efficacy in prognosis and risk stratification. In patients with no liver metastasis, Amp6_20q13.33 was a significant prognostic factor and an independent risk factor. The number of patients with or without Amp6_20q13.33 was balanced and therefore the conclusion is reliable. These results suggested that patients with Amp6_20q13.33 may have worse prognosis and poorer survival than those without Amp6_20q13.33, and this variation can be used as an independent marker for risk stratification, regardless of other influencing factors. In contrast, in patients with liver metastasis, Amp10_14q32.33, Del4_10q23.31 and TRIM77 mutations were significant prognostic factors, while TRIM77 was the only independent risk factor. These results suggested that all the three variations can be used for to predict the patient prognosis and survival, while only TRIM77 mutations can be used for risk assessment, regardless of other influencing factors. It should be noted that patients with TRIM77 mutations exhibited worse survival and poor prognosis than those without mutations in patients with liver metastasis, therefore, these patients may represented the population with worst prognosis in all patients involved in this study. Interestingly, TRIM77 mutations were also the one with significant frequency difference when patients with or without liver metastasis were compared (**Supplementary Table 1**). Patients with no liver metastasis had no TRIM77 mutations at all, while 17.24% of patients with liver metastasis had TRIM77 mutations. This observation suggested that TRIM77 mutations possibly only existed in the primary cancer tissues of patients with liver metastasis, and therefore was a potential indicator for liver metastasis.

TRIM77 belongs to tripartite motif (TRIM) family proteins, most of which have E3 ubiquitin ligase activities and have various functions in cellular processes, including intracellular signaling, development, apoptosis, protein quality control, innate immunity, autophagy, and carcinogenesis (19). TRIM proteins were suggested to be associated with oncogenic regulation, tumor-suppressive regulation, metastasis regulation and DNA repair (19). Many studies have highlighted the critical impact of TRIM protein family on CRC. Abundant expression of many TRIM proteins has been observed in CRC tissues and frequently correlated with poor survival of patients, while some TRIM members can act as tumor suppressors (20). For example, TRIM67 was suggested to activate p53 to suppress CRC initiation and progression (21), while TRIM47 was found to be up-regulated in CRC (22). TRIM52 was reported to promote CRC cell proliferation (23), and TRIM23 overexpression was suggested to be a poor prognostic factor and contributed to CRC carcinogenesis (24). Patients with higher TRIM24 expression had shorter survival time than those with lower TRIM24 expression (25), and TRIM14 promoted CRC cell migration and invasion (26), and TRIM59 facilitated the proliferation of CRC and promotes metastasis (27). Although these studies investigated the roles of some TRIM family proteins, the potential role of TRIM77 has never been reported so far. The fact that TRIM77 mutations were only present in primary cancer tissues of patients with liver metastasis suggested that TRIM77 mutations may correlate with subpopulations of primary cancer cells that migrate to liver, and therefore its mutations could be used as indicators for liver metastasis. Furthermore, TRIM77 mutations independently predicted poor prognosis, indicating highly malignant properties of metastatic tissues with the mutations. Therefore, TRIM77 mutations may be indicative for metastasis, prognosis and degree of malignancy.

The genetic prognostic factors have been widely investigated in previous studies (8, 9). However, most studies investigated the mutational landscape of liver metastatic tissue and correlated the key driver gene mutations with survival or prognosis. These studies found a series of prognostic mutations including TP53, KRAS, BRAF and PI3K (8, 9). In contrast, we performed analysis with the primary colorectal cancer tissue in this study and identified TRIM77 and a series of CNV changes as the prognostic factors, which was distinct from previous reports. Our study facilitated the prognosis prediction of patients with liver metastasis since primary cancer tissues are much easier to obtain than liver metastatic tissue. Prediction with primary tissue maximizes the chance that a patient can be assessed. In clinical practice, CRC patients should be tested for genetic variations as long as the primary cancer tissue is available. This not only facilitates the risk assessment and prognosis stratification, but also provides valuable information for their future therapy, especially when recurrence or metastasis occurs. Our findings in this study helped to stratify patients with or without liver metastasis before any therapy, and may influence the choice of therapeutic strategies. For example, surgery for patients with Amp6_20q13.33 may need to be more radical to achieve better survival, and patients should be more closely monitored within two years after surgery. More vigorous therapy may be applied to patients with TRIM77 mutations at the first line to achieve maximal remission in the first instance, and radical surgery may be considered whenever possible. These assumptions need to be tested and validated in larger studies in combination with current therapeutic strategies to achieve safe, reliable and effective treatment.

This study had some limitations. First, the prognostic factors identified in this study were derived from relatively small retrospective cohorts, and larger prospective validation is needed to confirm the effectiveness of the findings. Secondly, these findings from genomics methods should be validated by other methods at transcriptional (mRNA) and expressional (protein) levels. Thirdly, influence of genetic markers on recurrence and metastasis at multiple omics levels should be further investigated, and models for patient prognosis composed of multiple level markers should be established in future.

## Data Availability Statement

The datasets used and/or analysed during the current study are available from the corresponding author on reasonable request.

## Ethics Statement

The studies involving human participants were reviewed and approved by the Ethics Committee of The Third Xiangya Hospital of Central South University. The patients/participants provided their written informed consent to participate in this study.

## Author Contributions

HP, LS, and PC, designed the study. HP, GL, YB, XZ, LZ, CH, ZS, and SC recruited the patients, collected the samples, sent the samples for tests, and collected the clinical information and follow-up information for all patients. GL, SD, JZ, TH, YW, MX, and LS performed the sequencing test and sequencing data analysis for all samples. HP, GL, YB, and LS performed statistical analysis and made the figures and tables. HP, GL, LS, and PC wrote the manuscript. PC proof read the manuscript. LS submitted the manuscript. All authors contributed to the article and approved the submitted version.

## Funding

This study was supported by the National Natural Science Foundation of China [81872473(PC)];the Hunan Province Science and Technology Plan [2017SK2052 (PC)]. This study was also supported by the Special Funds for Strategic Emerging Industry Development of Shenzhen (Grant Number 20170922151538732), and the Science and Technology Project of Shenzhen (Grant Number JSGG20180703164202084). All funders did not participate in the study design, study implementation, data collection, data analysis, data interpretation and manuscript writing of the study.

## Conflict of Interest

GL, SD, JZ, TH, YW, MX, and LS are employees of HaploX Biotechnology, who performed the NGS sequencing and data analysis in this study.

The remaining authors declare that the research was conducted in the absence of any commercial or financial relationships that could be construed as a potential conflict of interest.

## Publisher’s Note

All claims expressed in this article are solely those of the authors and do not necessarily represent those of their affiliated organizations, or those of the publisher, the editors and the reviewers. Any product that may be evaluated in this article, or claim that may be made by its manufacturer, is not guaranteed or endorsed by the publisher.
